# Plant species with the trait of continuous flowering do not hold core roles in a Neotropical lowland plant‐pollinating insect network

**DOI:** 10.1002/ece3.7203

**Published:** 2021-02-10

**Authors:** Chelsea R. Hinton, Valerie E. Peters

**Affiliations:** ^1^ Department of Biological Sciences Eastern Kentucky University Richmond KY USA

**Keywords:** bees, butterflies, *Hamelia patens*, modularity, network theory, phenology

## Abstract

Plant–animal interaction science repeatedly finds that plant species differ by orders of magnitude in the number of interactions they support. The identification of plant species that play key structural roles in plant–animal networks is a global conservation priority; however, in hyperdiverse systems such as tropical forests, empirical datasets are scarce. Plant species with longer reproductive seasons are posited to support more interactions compared to plant species with shorter reproductive seasons but this hypothesis has not been evaluated for plant species with the longest reproductive season possible at the individual plant level, the continuous reproductive phenology. Resource predictability is also associated with promoting specialization, and therefore, continuous reproduction may instead favor specialist interactions. Here, we use quantitative pollinating insect–plant networks constructed from countryside habitat of the Tropical Wet forest Life Zone and modularity analysis to test whether plant species that share the trait of continuous flowering hold core roles in mutualistic networks. With a few exceptions, most plant species sampled within our network were assigned to the role of peripheral. All but one network had significantly high modularity scores and each continuous flowering plant species was in a different module. Our work reveals that the continuous flowering plant species differed in some networks in their topological role, and that more evidence was found for the phenology to support specialized subsets of interactions. Our findings suggest that the conservation of Neotropical pollinating insect communities may require planting species from each module rather than identifying and conserving network hubs.

## Graphical abstract

Plant species with longer reproductive seasons are posited to support more interactions compared to plant species with shorter reproductive seasons but this hypothesis has not been evaluated for plant species with the longest reproductive season possible at the individual plant level, the continuous reproductive phenology. Here, we use a quantitative pollinating insect–plant network constructed in tropical lowland wet forest and modularity analysis to test whether species that share the trait of continuous flowering hold core roles in mutualistic networks. Our work reveals that the continuous flowering plant species differed in some networks in their topological role, supporting specialized subsets of interactions more commonly, and that conservation of pollinating insect communities may require planting species from each module rather than identifying and conserving network hubs.

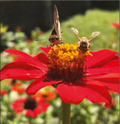

## 1 INTRODUCTION

The diversity of mutualistic plant–animal interactions in the Neotropics exceeds that of all other terrestrial regions globally (Fleming & Kress, 2013). It has been posited that the higher diversity of plant–animal mutualisms in the Neotropics is a result of the region's high spatio‐temporal predictability of fruit and nectar resources, and that these abundant resources can be attributed to the unique evolutionary history of the Neotropical flora, which includes (a) an Andean‐centered radiation of epiphytes, understory shrubs and palmetto‐like monocots and (b) an Amazonian‐centered radiation of canopy tree species and lianas (Fleming et al., 1987; Fleming & Muchhala, 2008). Within these two plant groups, certain plant species or genera should contribute substantially more to reducing resource patchiness owing to their asynchronous production of resources (Milton et al., 1982) or a longer reproductive season at the individual plant level (Gentry, 1974). Animal‐mediated seed dispersal and pollination are ecological processes that maintain global biodiversity patterns (Bascompte & Jordano, 2007; Bascompte et al., 2006; Bastolla et al., 2009). Thus, successful conservation and restoration efforts of Neotropical biodiversity overall requires a better understanding of the relationship between resource predictability and seed dispersal and pollination service provider communities (Hegland et al., 2009; Howe, 2016; Tylianakis et al., 2008).

Plant–animal science and mutualistic network theory predicts that plant species with longer reproductive seasons will support a higher number of animal species as well as more interactions in the network, and this trait should be associated with the role of network hubs in mutualistic networks (Carlo et al., 2007; Olesen et al., 2008; Tur et al., 2015; Waser et al., 1996; Yang et al., 2013). Network hubs are expected to play key organizational and structural roles in mutualistic communities, such that their loss from the network is expected to have the greatest overall effect on the risk of secondary extinctions owing to the loss of a high number of interactions (Mello et al., 2015; Olesen et al., 2007). The identification of plant species assigned core roles, that is, the topological roles of network hub and connector, in ecological networks is a priority in conservation ecology because of the potential that these species have in supporting biodiversity and ecosystem function (Cagnolo, 2018; Peters et al., 2016) yet data required to identify these species in each region from empirically derived local networks is costly and time‐consuming. However, if plant species that are assigned core roles in ecological networks share morphological or phenological traits, then this information can guide conservation and restoration action in locations where a local network has not been constructed.

While most plant species have temporally well‐defined phenology patterns, a few shrub and treelet species, in aseasonal environments, show steady‐state or continuous reproductive phenology strategies in which they produce resources daily over extended periods that span up to entire years (Gentry, 1974; Newstrom et al., 1994). Plant species with continuous resource production at the individual plant level are most diverse in lowland tropical wet, moist and humid forest types, however, even at peak richness they only comprise approximately 7% of all plant species (Bawa et al., 2003; Opler et al., 1980). The phenology shows a decrease in richness with altitude and is almost absent from lowland tropical dry forest types (Opler et al., 1980). Although a rare phenological trait, plant species with continuous resource production are ubiquitous throughout the Neotropical countryside, often planted as ornamentals, and therefore, if this trait is associated with core roles in mutualistic networks, then there is great potential to broadly implement their use as priority plant species for Neotropical biodiversity conservation and restoration. While evidence supports the role of an extended resource duration for supporting biodiversity in temperate systems (Olesen et al., 2008; Tur et al., 2015), continuous resource duration may instead promote specialization, as resource predictability is also posited to promote specialization (Betts et al., 2015; Borrell, 2005; Johnson & Steiner, 2000), with the degree of specialization increasing as the diversity of predictable resources increases (Fleming et al., 1987; Fleming & Muchhala, 2008). Thus, plant species with continuous resource production may behave similarly to plant species with extended resource production, accumulating more partners over time and playing core roles in mutualistic networks, or conversely promote specialization by providing temporally predictable resources. Reconciling the role of plant species with continuous resource production therefore is not only important for improving our theoretical understanding of how resource duration relates to the generalization–specialization spectrum and species roles in ecological networks but it also has important implications for how conservation practitioners and land managers approach conservation and restoration of mutualistic partners in environments where this phenology is found.

Pollinating insects are critical for maintaining biodiversity and food security in the tropics, which harbors the richest genetic warehouse of future pharmaceuticals and wild crops, as well as hundreds of cultivated crop species and potential nontimber forest products (Ashworth et al., 2009; FAOSTAT, 2018; Leakey, 2020). Despite this, tropical plant–pollinator networks remain limited in number and overrepresented by vertebrate pollinators (e.g., hummingbirds and bats) (Vizentin‐Bugoni et al., 2018). The development of pollinating insect–plant network studies in the megadiverse tropics is an urgent task given the current data‐deficiency coupled with the unprecedented rates of habitat modification and functional extinctions of interactions in the tropics (Barlow, 2018; Carreira et al., 2020). Here, we construct a pollinating insect–plant network from countryside habitat in the Tropical Wet forest Life Zone (Holdridge, 1967; Sanchez‐Azofeifa et al., 2002). Our goal in constructing the network was to empirically test if plant species that share the trait of continuous flowering will always be assigned to a core role in the network, that is, network hub or connector. We focused on countryside habitats of the Tropical Wet Life Zone because (a) Tropical Wet forest types have the highest diversity of plant species with the phenological trait of continuous flowering, and (b) countryside habitats, which includes smallholder farms and home gardens, represents the focal habitat for which our study is intended to recommend restoration and conservation action. We hypothesized that plant species with continuous flowering would support more interactions, and therefore hold a core role in the network (i.e., are network hubs or connectors) compared to plant species with shorter flowering seasons. We used the network‐level approach of modularity analysis to quantify plant species’ topological role in (a) the full and reduced network (i.e., all sampled periods across three years), (b) eight location‐based networks, (c) three consecutive networks (i.e., networks from the same months each year), and (d) two seasonal networks (i.e., comparing different months within the same year). Finally, focusing only on comparing the continuous flowering plant species to each other, we asked if they differed in the number of species or individuals of pollinating insects they could support.

## 2 METHODS

### 2.1 Study area

Our study took place in the southern Pacific lowlands of the Puntarenas Province of Costa Rica in the Osa Peninsula (OP; 8°N, −83°W). The OP contains 2.5% of the world's biodiversity and is home to the Corcovado National Park, which takes up the majority of region. The OP receives between 3,000 and 7,000 mm mean annual rainfall and mean temperatures of 24–26.5°C, and is comprised of Tropical Wet, Tropical Moist, and Premontane Wet forest types (Sanchez‐Azofeifa et al., 2002). Within the OP, we selected eight sampling locations along the main road that runs through the OP from Palo Seco (8°36′14″N, −83°26′57″W) to Rio Piro (8°24′11″N, −83°20′14″W; Figure S1).

### 2.2 Sampling design

As webs or networks become larger, insufficient sampling, as well as the risk of not detecting rare interactions increases (Basilio et al., 2006). Sampling over longer time periods, however, increases the chance that (a) potential partners in the network actually have nonoverlapping phenologies and (b) species with extended phenologies will have exaggerated generalization scores than what they have at any given time. Therefore, it is recommended that network properties and species topological roles be quantified within seasons but across years (i.e., in consecutive networks) rather than cumulative networks, that is, the entire year (Basilio et al., 2006). Since we were particularly interested in evaluating species topological roles and were including plant species with extended phenologies, we aimed to increase our resolution of within season interactions by (a) observing each plant individual for a 30‐min observation period, (b) conducting repeated sampling at all sampling locations within a season, and (c) sampling across eight sampling locations throughout the Osa Peninsula that had similar plant species compositions, habitat types, and surrounding land use types (see Section 2.3 for details). Seasonality in the Tropical Wet forest Life Zone is not well defined and consists of a six‐month rainy season (May–November) and a six‐month less rainy season (December–April). We collected data over a three‐week period during the middle of the rainy season (June–July) to ensure that all interacting partners were active during the entire study and to reduce the chance that the continuous flowering plant species would be assigned exaggerated generalization scores, increasing their likelihood of being assigned core roles in the network. In our study system, it is more likely that a plant species will become inactive (stop flowering) than that a bee species becomes inactive, since it is assumed that most tropical bee species are active throughout the year (Wolda, 1988). Three within‐rainy season consecutive networks were constructed, for the years 2017, 2018, and 2019. Four of the eight sampling locations were surveyed during 2017 and 2019, and six of the eight locations were sampled during 2018. Selection for which locations were surveyed each year depended on availability, for example, we were unable to survey the Osa Conservation location in 2019 because herbicide was applied broadly to the study area. Data were also collected across two of the eight sampling locations during a one‐week period in December 2017, to understand whether topological role of plant species differed between the rainy and less rainy seasons. Data were only collected in December 2017 owing to funding constraints. We conducted the study outside the peak flowering season (April–May) to help ensure that we collected data within a season where there would be a greater chance that continuous flowering plants would accumulate their true number of partners since fewer coflowering or other preferred resources would be available in the community. Finally, we also constructed location‐specific networks for five sampling locations from 2018 data, and for three locations from 2019 data, to ensure that topological roles were not location specific.

### 2.3 Sampling locations and pollinator sampling

**FIGURE 1:**
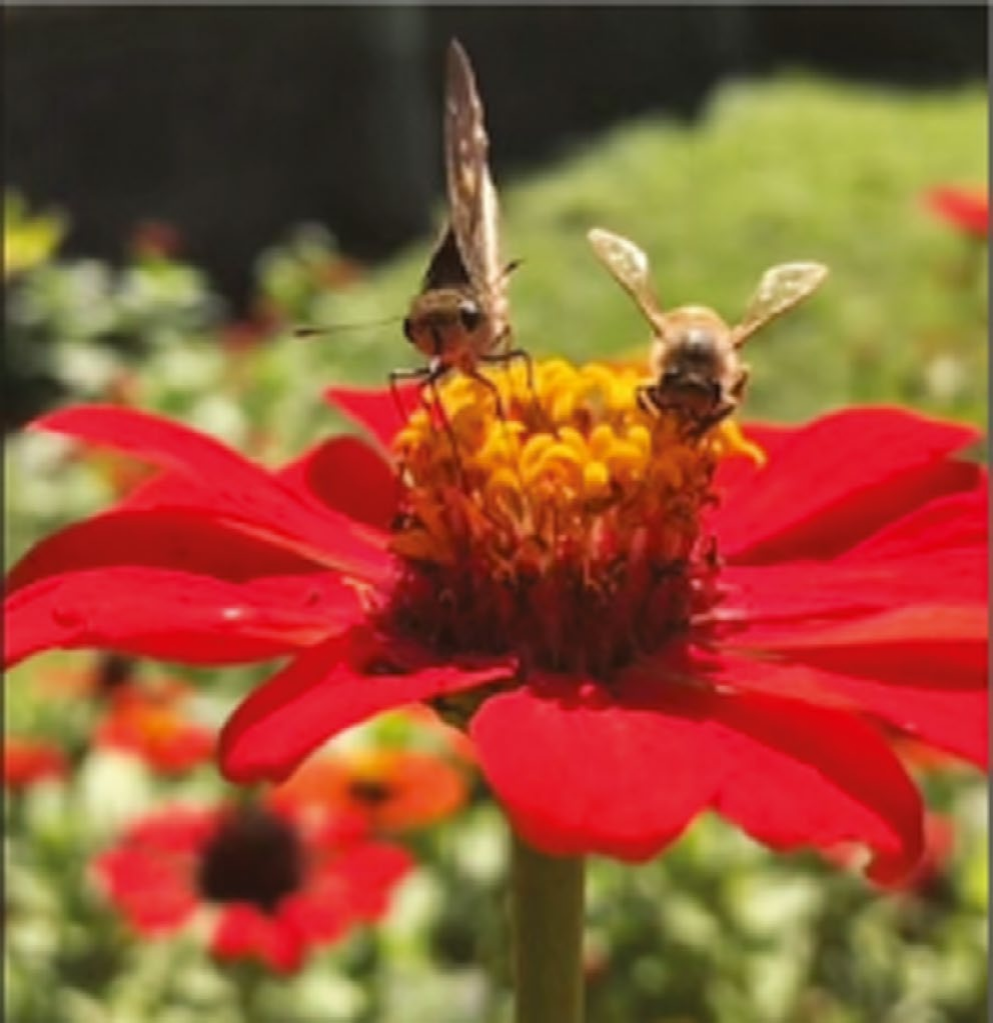
Photograph by Hancel Munoz. *Apis mellifera* (right) and Hesperiidae species (left) foraging on *Zinnia peruviana* in Osa Peninsula, Costa Rica

Throughout the study area, we located naturally occurring and planted individuals of six native shrub species that produce flowers daily, during all months of the year in the region. These continuous flowering shrub species were *Caesalpinia pulcherrima* (L.) Sw. (Fabaceae), *Conostegia subcrustulata* (Melastomataceae), *Hamelia patens* Jacq. (Rubiaceae), *Lantana camara* L. (Verbenaceae), *Stachytarpheta frantzii* Pol. (Verbenaceae), and *Turnera subulata* Sm. (Passifloracaea) (Table S1). Individuals of these six shrub species were abundant throughout the countryside habitats of the region. We focused on tropical countryside habitats rather than intact forest because our study aims to make management recommendations for conservation and restoration action for the various countryside habitats. Furthermore, pollinators are more readily sampled from open, sunny areas and within intact forest, naturally occurring individuals displaying a continuous flowering phenology typically only occur in forest edges and treefall gaps, making sampling these areas more logistically challenging. A total of eight sampling locations were selected throughout the Osa Peninsula to ensure that observed interactions were representative of the plant species across the tropical countryside and not just one particular location. Sampling locations varied in area sampled (from approximately 3 km to 5 km, following along roadsides) to ensure that each location included individuals of at least two of the six continuous flowering plant species. All eight sampling locations were similar in that they each included roadside, home garden and smallholder farm habitat types that included plant species with a continuous flowering phenology as well as coflowering plant species with a range of shorter flowering phenologies (Table S1). All eight sampling locations were located within a matrix of habitat types that included agriculture, secondary forest, and forest patches, but we only sampled from roadside, home garden, and smallholder farms in each location. Sampling locations were all >8 km from the closest border of Corcovado National Park. Plants sampled were either planted by landowners or were naturally occurring. We aimed to sample from each location 5–7 times a week throughout the study period, but that was not always feasible given logistical constraints and weather. The order of location per day was chosen at random. During each sampling period, each flowering plant species in the sampling location was observed during a 30‐min time interval and all butterflies and bees touching the plant's reproductive structures were captured and euthanized for later identification. Pollinator sampling was conducted daily from 08:00–15:00 hr daily except for during periods of heavy rain. Owing to a pilot study conducted in the study area during 2016, we targeted the collection of two insect taxa: bees (Hymenoptera: Apoidae) and butterflies (Lepidoptera), since they were the primary flower visitors (Figure 1; Table S2). Collected Lepidoptera were taken to the UGA field station in NW Costa Rica and were identified to species or genus by J. Montero and using the guides (DeVries, 1987; Glassberg, 2018). Bees were preserved in 70% ethanol and were exported to Eastern Kentucky University where they were identified to species, genus, or morphospecies using several keys: Michener (2000), Mawdsley (2017), Aguiar and Melo (2011), and Roubik and Hanson (2004). Centridini and Ceratinini bee species were identified using reference collections established by J. Pawelek and Dr. S. Rehan, respectively. For each continuous flowering plant individual, we also estimated floral abundance by counting the number of open flowers on 5–10 branches and then multiplying this number by the number of branches on the plant.

### 2.4 Data analysis

We assessed sampling completeness of the insect pollinator community and plant–pollinator interactions for the whole network, with all plant species pooled. Additionally, we assessed sampling completeness of the visiting insect pollinator assemblage for each plant species separately, only considering plants with ten or more samples (see Chacoff et al., 2012). To achieve this, rarefaction curves were generated to illustrate the accumulation of species (Figure S2a) or interactions (Figure S2b) as more individuals were collected. We extrapolated the asymptotic richness of species (*S*_E_) and interactions (*I*_E_) using a Chao estimator, which considers rare and unseen species (Chao, 1987), and then calculated what percentage of the expected richness was detected within our study for each level of sampling completeness analysis (see Chacoff et al., 2012). Analyses were conducted using the “vegan” package (Oksanen et al., 2019) in R (R Development Core Team, 2019).

A quantitative or weighted plant–pollinator interaction matrix was created from visitation data for (a) the full network pooling all the data from each sampling location and across all years and seasons (25 plant and 171 pollinator species); (b) a reduced matrix which excluded species that were found at fewer than two sampling locations (14 plant and 87 pollinator species); (c) eight location‐specific networks; (d) three consecutive networks; and (e) two seasonal networks. The three consecutive networks were matrices of interactions pooled across all sampling locations for that year but separated into years for June‐July data only (2017, 2018, and 2019). The two seasonal networks were matrices of interactions pooled across sampling locations but separated into seasons: June–July 2017 (rainy season) and December 2017 (dry season). Cell values in each matrix indicate the frequency of interactions between species pairs, and cells with zeros indicate no interaction.

Quantitative modularity computes modules for weighted bipartite networks, with each module defined by species having more interactions within the module than among modules (Beckett, 2016; Dormann & Strauss, 2014). Modularity therefore refers to species sets that interact more frequently with each other and is the result of some degree of specialization in species interactions (Olesen et al., 2007; Watts et al., 2016). Quantitative modularity analysis was conducted on each weighted network to (a) quantify modularity and (b) identify modularity roles for all species in the lower trophic level (i.e., all plant species) using the quantitative DIRTLPAwb + algorithm (Beckett, 2016) in R “bipartite” package version 2.13 (Dormann et al., 2009). As network size and sampling intensity can influence the observed modularity value, a null model comparison approach was used to standardize the observed value ($Z_{Q}=\frac{Q_{\mathrm{observed}}‐{\bar{Q}}_{\mathrm{null}}}{\sigma_{Q_{\mathrm{null}}}}$), where a Z*_Q_* > 2.0 indicates a significantly modular structure (Carstensen et al., 2016; Saunders & Rader, 2019). The mean null modularity (${\bar{Q}}_{\mathrm{null}}$) was obtained from 100 quantitative null models using the *vaznull* method (Vázquez et al., 2007).

Modularity roles for all plant species were identified using critical thresholds of among‐module connectivity (*c*) and within‐module connectivity (*z*), where c refers to how evenly links are distributed within and across modules and z refers to the number of links within the module (Olesen et al., 2007; Watts et al., 2016). We calculated weighted c‐ and z‐values using interaction strength (species strength) instead of species degree because species strength is more appropriate for quantitative networks while the use of species degree was defined for use with binary networks (Dormann & Strauss, 2014; Watts et al., 2016). We ran 100 null models and employed 95% quantiles to establish objective critical thresholds of *c*‐ and *z*‐values (Dormann & Strauss, 2014). Next, weighted *c*‐ and *z*‐values were calculated for all lower trophic level species in the observed network. Using the critical thresholds, plant species were then assigned into one of four network roles, peripherals, connectors, module hubs, and network hubs. Peripherals are species that have weaker interactions with partners and primarily interact within their module, with species *c‐* and *z*‐values falling below both critical thresholds. Connectors are species that bind modules, supporting stronger interactions among modules than within modules, with species *c‐*values > *c*_critical_ and species *z‐*values < *z*_critical_. Module hubs are defined as those species having a high number of interactions within the module that they occur such that species *c‐*values < *c*_critical_ and species *z‐*values > *z*_critical_. Finally, network hubs are species that have a high number of interactions both within their own module, as well as among the other modules, with species *c‐*values > *c*_critical_ and species *z‐*values > *z*_critical_).

To evaluate whether the abundance of butterflies and bees and the number of butterfly and bee species collected per sample differed among the continuous flowering plant species, we used generalized linear and linear mixed models. Butterfly and bee abundance and butterfly and bee species richness per sample were modeled separately as the response variables, with each model containing plant species identity as the fixed effect and sampling location as the random effect. To evaluate the effect of the individual continuous flowering plant's floral abundance on butterfly and bee abundance and butterfly and bee species richness per sample, we used generalized linear and linear mixed models. Butterfly and bee abundance and butterfly and bee species richness per sample were modeled separately as the response variables, with the individual continuous flowering plant's floral abundance as the fixed effect and sampling location as the random effect. A negative binomial error distribution was used for the two species richness models, while abundance models were log transformed to meet the conditions of normality. Likelihood ratio tests were used to test the significance of fixed effects in each model. Tukey's post hoc comparisons in the package “multcomp” R package were used to delineate species‐specific differences among continuous flowering plant species for each response variable (Hothorn et al., 2008). All statistical analyses were performed using R version 3.5.3 (R Development Core Team, 2019).

## 3 RESULTS

**FIGURE 2:**
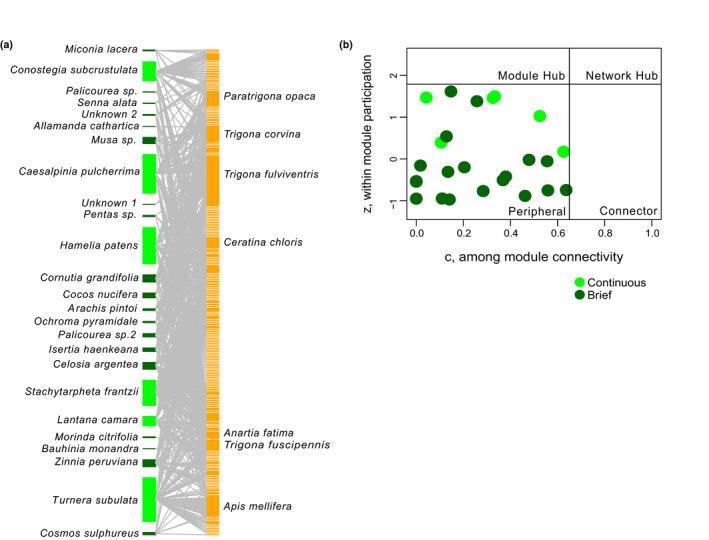
(a) Full bipartite network from Osa Peninsula, Costa Rica. Continuous flowering plant species are shown in light green, and all other plant species are shown in dark green. Bee and butterfly species are shown in gold. (b) Full network roles assigned using quantitative modularity analysis. Quadrants are labeled based on the weighted *c*‐ and *z*‐values

A total of 5,703 interactions between 25 plant species and 171 pollinator species, including a total of 4,894 interactions representing 87 bee species and 809 interactions representing 84 butterfly species, were observed across all sampling locations and years (Table 1, Table S2, Figure 2a). In 2019, 2,783 of the total 5,703 interactions were added to the network, but this resulted in the addition of only 6 new bee species to the network. Extrapolated asymptotic richness estimated 247 ± 27.8 pollinator species within the community, suggesting we captured approximately 69% of the expected species within the study area (Figure S2a). A total of 506 unique plant–pollinator interactions were observed within our study, and extrapolated asymptotic richness estimated 921 ± 73.3 possible interactions, suggesting we detected 55% of the expected interactions (Figure S2b). The percentage of the estimated interactions detected varied across plant species within our study (Table S3).

**TABLE 1:** Number of species collected from the Osa Peninsula, Costa Rica, representing bee and butterfly families

Order	Family	Number of species
Hymenoptera	Apidae	66
	Halictidae	19
	Megachilidae	2
Lepidoptera	Hesperiidae	37
	Lycaenidae	6
	Nymphalidae	24
	Papilionidae	2
	Pieridae	12
	Riodinidae	3

The full network, constructed from all observed interactions, was found to be significantly modular (*Q* = 0.48, *Z*_Q_ = 107.48; Table 2 and Figure S3). All sampled plants were assigned to the role of peripheral in the full network (Figure 2b). The reduced network included 5,303 interactions and was also found to be significantly modular (*Q* = 0.47, *Z*_Q_ = 113.26; Table 2). Only one plant species, the continuous flowering plant species *Hamelia patens*, was assigned the topological role of connector. All other plant species were assigned the role of peripheral in the reduced network.

**TABLE 2:** Network‐level information for each network from the Osa Peninsula, Costa Rica

Network	Plants	Pollinators	Visits	Modules	*Q*_observed_	*Q*_adjusted_	*Z*_Q_
Full	25	171	5,703	6	0.48	0.39	107.48
Reduced	14	87	5,303	6	0.47	0.40	113.26
2017 Dry Season	8	36	327	5	0.48	0.28	13.62
2017 Rainy Season	7	70	756	5	0.46	0.25	20.68
2018 Rainy Season	15	101	1837	8	0.55	0.40	47.85
2019 Rainy Season	19	105	2,783	5	0.48	0.37	56.81
Finca Kobo/Palo Seco	10	34	461	7	0.53	0.29	13.71
Osa Conservation	8	60	532	6	0.61	0.39	19.67
Playa Sandalo	4	16	60	4	0.29	0.07	0.97
Canaza	8	39	347	6	0.61	0.40	17.80
Mata Palo	4	29	130	4	0.62	0.27	6.13
Playa Blanca*	2	22	113	2	0.44	0.15	2.67
Rincon*	9	35	269	5	0.50	0.26	9.88
Puerto Jimenez*	12	73	1925	3	0.47	0.37	56.15

Note

Location‐specific networks with an asterisk (*) indicate data from 2019 were used for analysis

**FIGURE 3:**
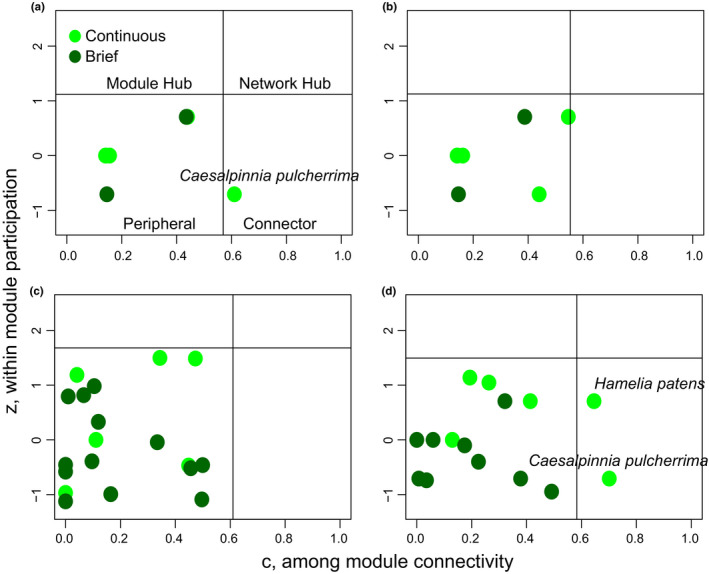
Consecutive and seasonal networks. Seasonal variation in network topology was assessed by comparing the rainy (a), and the less rainy season (b) of 2017. Topological role differences between years was assessed by comparing the rainy seasons of (a) 2017, (c) 2018, and (d) 2019

The rainy season network of 2017 was comprised of 756 interactions, 7 plant species, 33 butterfly species and 37 bee species, and had 5 modules (Table 2). The dry season network of 2017 was comprised of 327 interactions, 8 plant species, 36 bee species, no butterfly species, and had 5 modules (Table 2). One plant species, the continuous flowering plant species *C. pulcherrima*, was assigned the topological role of connector in the rainy season of 2017. All other plant species were assigned the role of peripheral in the rainy season 2017 network, and all plant species were assigned the role of peripheral in the dry season 2017 network (Figure 3a,b).

The rainy season network of 2018 comprised a total of 1,837 interactions, 15 plant species, 67 bee species and 34 butterfly species, and had 8 modules (Table 2). Most plant species were peripherals; however, two plant species were assigned the topological role of connector, continuous flowering species, *H. patens* and *C. pulcherrima* (Figure 3c). The rainy season network of 2019 comprised a total of 2,783 interactions, 19 plant species, 45 bee species and 60 butterfly species and had 5 modules. All plant species in the network were assigned peripheral roles (Figure 3d). All rainy and dry season networks were found to be significantly modular (*Z*_Q_ > 2.0; Table 2).

All location‐specific networks were significantly modular except one (Table 2). Only one plant species for one of the location‐specific networks was assigned the topological role of connector, the continuous flowering plant species *C. pulcherrima* in the Palo Seco network. Two plant species were assigned the topological role of module hub in the Puerto Jimenez network, each for a separate module in the network. Both module hubs were continuous flowering plant species, that is, *Turnera subulata* and *C. pulcherrima*. All other plant species in the remaining six sampling locations were assigned the topological role of peripheral. No plant species were assigned the topological role of network hub in any of the fourteen constructed networks described above.

**FIGURE 4:**
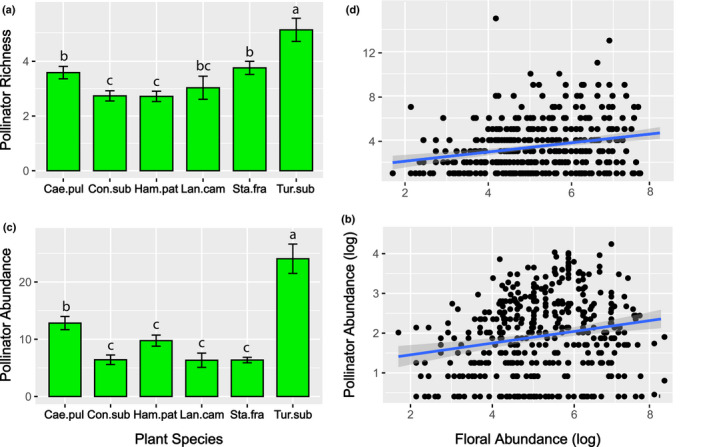
The mean and standard error for (a) bee and butterfly richness and (c) abundance comparing six continuous flowering plant species: *Caesalpinia pulcherrima* (Cae.pul), *Conostegia subcrustulata* (Con.sub), *Hamelia patens* (Ham.pat), *Lantana camara* (Lan.cam), *Stachytarpheta frantzii* (Sta.fra), and *Turnera subulata* (Tur.sub). The relationship between the floral abundance of an individual continuous flowering plant and bee and butterfly abundance is shown in (b) and (d)

Continuous flowering plant species differed significantly in the number of butterfly and bee species per sample (*X* ^2^ = 51.07, *p* < .0001; Figure 4a). Post hoc comparisons show *Turnera subulata* had significantly more species‐rich samples ($\bar{x}$ = 5.15 ± 0.42) compared to all other continuous flowering plant species. Butterfly and bee abundance differed significantly between continuous flowering plant species (*X* ^2^ = 75.80, *p* < .0001) with *T. subulata* also having more interactions per sample ($\bar{x}$ = 24.06 ± 2.56) than all other species (Figure 4c). Although *H. patens* and *C. pulcherrima* had more interactions than the remaining continuous flowering plant species ($\bar{x}$ = 9.76 ± 0.97 and $\bar{x}=$ 12.83 ± 1.15, respectively), only *C. pulcherrima* was statistically higher than the other species. An individual continuous flowering plant's floral abundance had a significantly positive effect on butterfly and bee species richness per sample (*X* ^2^ = 25.79, *p* < .0001) and butterfly and bee abundance per sample (*X* ^2^ = 22.60, *p* < .0001). Individual plants with more flowers were visited by more butterfly and bee species and more butterfly and bee individuals per sample (Figure 4b,d).

## 4 DISCUSSION

Our results show that plant species with a continuous flowering season were not more connected in the network; in fact, most plant species with this phenology were consistently assigned peripheral roles alongside brief, annual flowering plants. This finding is novel for community ecology, which has previously demonstrated that longer resource production should always be associated with higher levels of generalism (Martin‐González et al., 2012; Waser et al., 1996). The longest resource production at the individual plant level, continuous flowering, and fruiting, however, may have a functionally different role in mutualistic networks, instead promoting specialization owing to their small stature as understory plants and small floral displays which render them unable to support the energetic needs of a generalist assemblage of pollinators (Borrell, 2005). This finding has important implications for pollinator restoration and conservation in countryside habitats of the Tropical Wet forest Life Zone, where plant species with this phenology are the most diverse, because restoration of ecosystem function may require restoration of each module rather than the generalist core.

In the network, although pollinators were quite connected (Figure 2), subsets of stronger interactions formed modules that were characteristic (Figure S3). For example, the purple flowering *Stachytarpheta frantzii* formed a module with Euglossine bees and Hesperiidae butterflies, where 89.7% of all Euglossine bees and 51.4% of all Hesperiidae butterflies were collected from a *S. frantzii* individual. The continuous flowering shrub species, *H. patens,* which was a connecter in two networks, formed another module of the network, by having strong associations between bee species from the tribe Ceratinini (55% of all Ceratinini were collected from *H. patens*) as well as with the most generalist bee species in the network, *Trigona fulviventris* (42% of all *T. fulviventris* were collected from *H. patens*). One final example is the module formed by the association between the continuous flowering plant species, *Conostegia subcrustulata* and the bee species *Augochloropsis ignita* and *Melipona fasciata*, which is an important honey‐producing Apis‐sized eusocial stingless bee. All *A. ignita* individuals and >95% of all *M. fasciata* individuals were collected from *C. subcrustulata* flowers. Thus, our results support the idea that when pollinator species can rely on specific resources that are temporally predictable, specialization is more common (Johnson & Steiner, 2000).

In most published bee–plant interaction studies, including from higher elevations in the tropics, *Apis mellifera* is considered a super generalist with the highest number of interactions (Ricketts, 2004; Souza et al., 2018; Watts et al., 2016). Our network, in contrast, found a social, stingless bee (Tribe Meliponini), *Trigona fulviventris,* to have more interactions than *A. mellifera. Trigona fulviventris* was observed visiting nearly all plant species within our study, but most strongly interacted with *H. patens* (Figure S3). Only two plant species, the continuous flowering plant species *H. patens* and *C. pulcherrima,* were assigned the topological role of connector, and each in only two and three of the fourteen networks, respectively. One brief flowering plant species, *Isertia haenkeana*, came the closest to being assigned a connector species in the full network. All three plant species had strong associations with generalist stingless bee species (Tribe Meliponini), potentially suggesting that plant species supporting generalist stingless bee species are more connected in the network compared to plant species that support other pollinating insect groups or bee tribes. The inclusion of brief, episodic flowering tree species with larger floral displays in the network would likely increase the importance of *Apis mellifera* in the network, as *A. mellifera* visits tend to be more tightly linked to floral density compared to other bee species in the tropics.

Phenological overlap is thought to play an important role in structuring plant–pollinator interactions, meaning that species with longer phenologies will interact with more partners. This is a simple, intuitive prediction that has received considerable support (Martin‐González, 2010; Martin‐González et al., 2012; Olesen et al., 2008; Tur et al., 2015; Waser et al., 1996; Watts et al., 2016), including from studies using network theory approaches (Vázquez et al., 2009; Vizentin‐Bugoni et al., 2018; Yang et al., 2013). Although our study is not the first to find a mismatch between phenophase duration and generalism using a network approach (e.g., Russo et al., 2013), our study is the first to evaluate the hypothesis for the continuous flowering phenology and in the Tropical Wet forest Life Zone. A central concept of Waser et al.'s (1996) hypothesis is that partners will be accumulated over time, and from the perspective of a plant species that produces flowers during all days of the year, this means that either the phenology of the pollinators is seasonal or the pollinators will switch resource use when preferred plant resources are no longer available, otherwise temporal overlap of the plant and pollinator partners would be constant. In Tropical Wet forest Life Zone habitat types, plant species may not accumulate partners over time in the same way that temperate plant species with extended resource production do if pollinator phenologies are less well defined (i.e., pollinators are active all year round). While records of the phenology of most tropical insect species are nonexistent and at best fragmentary (e.g., Michener & Eickwort, 1966; Sakagami & Laroca, 1971), it is assumed that bee and butterfly species in the tropics are active all year round. Eusocial bee species of the tribes Meliponini, Apini, and Bombini have perennial colonies and are well documented to be active all year round (Jarau & Barth, 2008; Roubik & Hanson, 2004). Bee species of these three tribes represented 65%, 11%, and 1%, respectively, of all observed interactions. Bee species in the tribe Meliponini were by far the most abundantly collected bee species in the network and during our study we collected 23 of the 26 species known to occur on the Osa Peninsula (Jarau & Barth, 2008). Therefore, if pollinating insects are active all year round in the tropics and continuous flowering plant species are active all year round as well, then these plant species should not be able to accumulate more partners over time, unless pollinators are switching resource use between a preferred floral resource and the continuous flowering plant species.

In a similar vein, we conducted our study outside of the peak flowering season in an effort to ensure that continuous flowering plant species would be more likely to accumulate their true number of partners and reduce the chance that preferred floral resources were drawing bee and butterfly species away from the continuous flowering plant species. Our data indicate that we likely did not miss bee and butterfly species because they were not phenologically active or visiting other preferred floral resources that we did not sample. For example, we collected 23 of 26 Meliponini species found to occur in the Golfo Dulce region, a larger region that includes our study area as well locations on the other side of the Golfo Dulce, including Golfito and surrounding areas (Jarau & Barth, 2008). Since the work of Jarau and Barth (2008) represents the only comprehensive survey for any bee or butterfly tribe in the area, future work could aim to test the validity of the assumption that other bee and butterfly species are active throughout the year and were not visiting other preferred floral resources by including more months of the year and thus more plant species in the network.

Future work should aim to evaluate the role of continuous resource production in other ecosystems, or land use types, as it is also possible that our finding that the majority of plant species are peripherals in the network is an artifact of the characteristics of the study area. For example, specialization is assumed to increase with species richness as well as with decreasing latitude and elevation, although this is still somewhat controversial (Hoiss et al., 2013; Schleuning, 2012; Trøjelsgaard & Olesen, 2013) and, therefore in Tropical Montane and Pre‐montane Life Zones where there are typically fewer than 3 continuous flowering plant species, the phenology may function differently in the community. Land use types within the Tropical Wet Life Zone, with a diverse assemblage of plant species that provide floral resources continuously may foster plant–pollinator interactions with higher levels of specialization and more modular structure compared to other global regions (Spiesman & Gratton, 2016). Highly modular networks have also been found in protected systems where the loss of specialized interactions has not yet occurred, and it is assumed that modularity protects communities within anthropogenic landscapes from species loss (Carreira et al., 2020; Ramos‐Robles et al., 2018). The widespread use of several different species of native, continuous flowering shrubs as ornamentals throughout the tropics may contribute to favoring modularity even in the anthropogenic landscape that we sampled. Our study was conducted in tropical countryside habitats because these habitat types have the potential to be managed throughout the tropics for pollinator restoration and conservation (e.g., Hopwood, 2008; Peters et al., 2012). The conservation of pollinators in smallholder farms can also increase food security for subsistence farmers who have a higher dependence on free pollination services (Ashworth et al., 2009). Undoubtedly, because highly modular networks have also been reported for networks with non‐native species included (Vizentin‐Bugoni et al., 2019) and species roles within the network are expected to change with disturbance level (Carreira et al., 2020), there is still an urgent need to study pollinating insect–plant networks in protected tropical forests and in the absence of introduced species in an effort to develop best management practices for complete ecological restoration of tropical forest community structure and function (Harvey et al., 2017; Raimundo et al., 2018).

Ecological networks have been described as temporally dynamic, changing the inner details from year to year, but maintaining broad level structural attributes (Alarcón et al., 2008; Chacoff et al., 2017; Petanidou et al., 2008). Regardless of the size, modularity remained consistently high across all subsets of our full network (Table 2). Plant species network roles were different in some subsets of the full network but almost consistently all plant species were assigned as peripherals with the exception of five of fourteen networks (Figure 3). Network differences may reflect which plant species were flowering in the study area during the sampling period, the impact of rainfall or drought on pollinator populations, or the high temporal variability in interaction identity that is posited to be prevalent in plant–animal interaction networks (Dupont et al., 2009; Petanidou et al., 2008).

While it is generally widely agreed upon that the community‐level approach of network analysis improves upon species‐level assessments in conservation science (Biella et al., 2016; Tylianakis et al., 2010), there have been two distinct approaches used in the literature to select priority species for conservation initiatives. The first approach uses a number of different species‐level and network‐level measures such as degree and normalized degree, interaction strength and species strength, closeness centrality and betweenness centrality, to evaluate or rank species (Campbell et al., 2019; Russo et al., 2013). This approach can often result in substantially different rankings of species, and little information is available to guide practitioners on the “best” measure for conservation decision‐making. The second approach, modularity analysis, identifies core species directly by assigning topological roles to species that indicate the degree to which the species supports other species in the network via both direct and indirect interactions (Biella et al., 2016; Carreira et al., 2020; Guimerà et al., 2007). This approach, however, assumes (a) that the most important species in the network will be assigned into core roles and (b) that species with core roles are those responsible for promoting stability in the network (Kaiser‐Bunbury & Blüthgen, 2015). However, not all studies using modularity analysis find species assigned into each core role (Watts et al., 2016, this study) and recent theoretical and experimental work has shown that network stability may be fostered by specialized interactions instead of generalized interactions in some ecosystems (Benadi et al., 2013; Hoiss et al., 2015). Theoretically, both approaches outlined above should produce the same results, with modularity analysis somewhat “synthesizing” some of the species‐level scores to assign species roles. For example, using modularity analysis, Biella et al., (2016) found that z‐ and c‐scores were highly correlated with the species‐level metrics of partner diversity and degree. Therefore, network hubs are expected to be the most generalist species in the network, having a high number of partners and links (Biella et al., 2016; Martin‐González, 2010). If we had used the first approach in our study, we would have concluded that based on degree or normalized degree that there was high variation in the degree of generalism among plant species, with some continuous flowering plant species only scoring slightly higher (i.e., more generalist) than plant species with briefer flowering phenologies (Table S4) while betweeness and closeness centrality measures ranked the continuous flowering plant species in a very different order, with at least two of the continuous flowering plant species scoring as low as plant species with briefer flowering phenologies, indicating very low connectivity among all the nodes in the network for these species. It is noteworthy to add that using this approach, we would have found that *Zinnia peruviana*, a native, herbaceous plant species that flowers from April‐October, had the same level of generalism (degree and normalized degree) as the continuous flowering plant species, *Hamelia patens*, but *H. patens* had the highest level of connectivity (betweeness and closeness centrality) of all plant species, while *Zinnia peruviana* had one of the lowest measures of connectivity (Table S4). In addition, *Stachytarpheta frantzii* had a remarkably high level of generalism, with over twice as many pollinating insect species supported than the next highest ranking plant species, but showed almost no connectivity in the network (Table S4). In assigning species into modularity roles, using modularity analysis, different measures of generalism, that is, degree and centrality measures, are quantitatively combined into the context of the network itself. For example, if we had not used modularity analysis, we would not have observed that although some plant species with continuous flowering support a very high number of pollinating insect species and some other plant species with continuous flowering are important to the connectance of the network, none of the species consistently fulfill core roles of the network, that is, network hubs or connectors. Since network hubs, connectors, and module hubs are posited to promote stability (Kaiser‐Bunbury & Blüthgen, 2015), this finding may imply that the loss of any plant species from our network would have the same, minimal impact on the full network.

From an applied perspective, it was our goal to identify a shared trait of plant species holding key organizational roles in Neotropical pollinating insect–plant networks as a first step toward identifying priority plant species that could be used broadly for pollinator conservation and restoration throughout the Neotropics, and with substantial empirical support from other regions, we selected the duration of the flowering season as the plant trait to be tested. Instead, we found that when resource duration is of the longest timespan possible at the individual plant level, the continuous phenology, then smaller subsets of the pollinator community interact more frequently with each different continuous flowering plant species forming modules in the network, often with no plants binding the network (connectors) or acting as super‐generalists (network hubs). This result implies that recovery and stability of countryside habitats in the Tropical Wet forest Life Zone may therefore depend on ensuring that plant species representing each module are retained or included in the landscape, an approach which differs from current strategies that focus on the inclusion of a generalist core.

The majority of plant–pollinator network studies conducted in the Neotropics have used subsets of the full network, or partial networks, as it is particularly challenging to sample the entire community both temporally and spatially in the aseasonal and megadiverse tropics (Vizentin‐Bugoni et al., 2018). Most partial plant–pollinator network studies in the tropics focus on vertebrate pollinators while most comprehensive studies were conducted in ecosystems such as alpine or coastal forests that are more easily sampled and less structurally complex than tropical lowland humid forests (Campbell et al., 2019; Vizentin‐Bugoni et al., 2018; Watts et al., 2016). Because bees are the most effective pollinators, and their conservation is essential for protecting pollination services in the Neotropics (Ashworth et al., 2009), we chose to subset our network to focus on pollinating insects and plants, and further subsetted the network to focus on the shrub, treelet, and ornamental plant community. Protecting pollinators in the tropical countryside has important implications for food security. We also subsetted the network temporally by sampling plants over a smaller time scale than the full season of a continuous flowering plant species (i.e., the entire year). Subsetting the network made the study more feasible and increased sampling resolution for rare interactions (Jordano, 2016). The validity of our conclusions, however, will require further work. For example, does modularity role change over time and within the year for continuous flowering plant species? We sampled outside the period of peak flowering, which usually occurs just prior to the rainy season in the study area, because our goal was to understand whether continuous flowering plant species hold core roles during all months of the year, including during times of resource scarcity. Continuous flowering plant species should be more likely to have an important role for pollinators during times of resource scarcity (Menz et al., 2011) and our finding that each species was assigned the role of peripheral outside of peak flowering makes it unlikely that during peak flowering continuous flowering plant species would be assigned a more important role; however, further research is needed to eliminate this possibility. Additional work could also include vertebrate pollinators, or other months in the network to evaluate whether these additional components of the network change our conclusions. Furthermore, new technologies for sampling pollinating insects from tropical forest trees using DNA sequencing could be used to include more tree species in the network (Vizentin‐Bugoni et al., 2018). Our results highlight the urgent need for more work in the tropics to (a) understand how more intermediate reproductive phenophases support pollinators, (b) identify priority plant species (i.e., network hubs and connectors) for insect pollinators throughout the tropics, and (c) test other plant traits hypothesized to be associated with generalism, such as an open, accessible flower structure. For example, using a network approach, the plant family Malphigiaceae has been identified recently as containing priority plant species for bees in other ecosystem types of the Neotropics (Campbell et al., 2019). Future efforts could use modularity analysis to evaluate the potential of Malphigiaceae and other plant species, such as the two species that were assigned the topological role of connector in some of the networks in this study (i.e., *Hamelia patens* and *Caesalpinia pulcherrima*) to be assigned core roles or be assigned core roles more consistently in other tropical habitats and life zones, and in mutualistic networks where more tree species are included.

## 5 CONCLUSION

Our results suggest that conservation efforts for pollinators in countryside habitats of the Tropical Wet forest Life Zone may require the inclusion of plant species from different network modules rather than focusing on protecting a core of network generalists or ensuring that a source of nectar or pollen is available throughout the year, two approaches which have gained momentum in temperate systems (Martin‐González, 2010; Menz et al., 2011; Pocock et al., 2012; Ramos‐Jiliberto et al., 2009). Since the trait of continuous flowering was not associated with core roles in the network, our study provides evidence that caution should be exercised when using the trait of phenological breadth to infer a plant species benefit to the consumer community. Rather than having an overlapping functional role in supporting pollinating insects, each continuous flowering shrub species may have an essential, complementary role that should be considered when implementing conservation and restoration strategies targeted to protect insect pollinator communities in tropical countryside habitats.

## CONFLICT OF INTEREST

We declare there are no known competing or conflicting interests.

## AUTHOR CONTRIBUTION

**Chelsea R. Hinton:** Data curation (equal); Formal analysis (lead); Methodology (equal); Validation (equal); Visualization (equal); Writing‐original draft (lead); Writing‐review & editing (equal). **Valerie E. Peters:** Conceptualization (lead); Data curation (equal); Formal analysis (supporting); Funding acquisition (lead); Methodology (equal); Resources (lead); Validation (equal); Visualization (equal); Writing‐review & editing (equal).

## Supporting information

Figure S1

Figure S2

Figure S3

Supplementary Material

## Data Availability

All data included in this study are available from the Dryad Digital Repository at https://doi.org/10.5061/dryad.sqv9s4n32.
