# Supplementary material for: Plant species with the trait of continuous flowering do not hold core roles in a Neotropical lowland plant‐pollinating insect network
Source: Ecol Evol. 2021 Feb 10;11(5):2346–59. doi: 10.1002/ece3.7203 (PMC7920781; doi:10.1002/ece3.7203)
Supplement: Supplementary file 4 — Supplementary Material [file ECE3-11-2346-s004.docx]

**Appendix**

**Table S1**. Lists all plant species sampled within our network and a brief description.

| **Plant Species** | **Family** | **Phenology** | **Brief Description** | **Sources** |
| --- | --- | --- | --- | --- |
| *Allamanda cathartica* | Apocynaceae | Unknown | Large, yellow, bell-shaped flowers |  |
| *Bauhinia monandra* | Fabaceae | Unknown | Treelet with large, orchid-like, pale pink and white flowers with some dark pink striping |  |
| *Arachis pintoi* | Fabaceae | Mainly in May to July, Sometimes continuous | Herbaceous, trailing plant with small yellow flowers. | Frankie et al. 2013, |
| *Caesalpinia pulcherrima* (L.) Swartz | Fabaceae | Continuous | Shrub or tree; flowers are yellow to orange-red, styles and stamens protrude from the flowers. | Janzen and Liesner 1980 |
| *Celosia argentea* Cav. | Amaranthiaceae | Unknown for the Neotropics | Herbaceous, with numerous small pink-green flowers forming a spiked inflorescence. |  |
| *Cocos nucifera* | Arecaceae | Continuous | Tree with small, highly abundant, pale yellow flowers growing in spike-like inflorescences | Thomas and Rajkumar 2013 |
| *Conostegia subcrustulata* (Beurl.) Tr. | Melastomataceae | Continuous | Shrub, roughly 2 meters tall. Large terminal inflorescences of very small, open, pale pink flowers. | Croat 1978, Kriebel 2016 |
| *Cornutia grandifolia* (Schlecht. and Cham.) Schau. | Lamiaceae | Mid-rainy season | Small trees with large, terminal clusters of pale blue-purple flowers. | Janzen and Liesner 1980, Enquist and Sullivan 2001 |

| **Plant Species** | **Family** | **Phenology** | **Brief Description** | **Sources** |
| --- | --- | --- | --- | --- |
| *Cosmos sulphureus* L. | Asteraceae |  | Tall herbaceous annual, with yellow or orange flowers. |  |
| *Hamelia patens* Jacq. | Rubiaceae | Continuous | Shrubs or small tree, red-orange tubular flowers. | Frankie et al. 1974, Croat 1987 |
| *Isertia haenkeana* D.C. | Rubiaceae | Flowering mostly early in the rainy season | Small tree or shrub, long tubular yellow flowers arranged in a stout panicle. Flowers have a flash of deep red on the inside. | Croat 1978 |
| *Lantana camara* L. | Verbenaceae | Continuous | Shrub with clustered, orange-red to yellow flowers in a umbel-like inflorescence. | Croat 1978 |
| *Miconia lacera* (Bonpl.) Naud | Melastomataceae | March-August | Shrub with small, white to pale pink flowers. | Croat 1978 |
| *Morinda citrifolia* | Rubiaceae | Unknown | small tree or shrub with small white, five-lobed flowers | Singh et al. 2006 |
| *Musa sp.* | Musaceae | Asynchronous at the population level | Tall and herbaceous, with a large purple bracht that conceals pale yellow-purple flowers. |  |
| *Ochroma pyramidale* (Cav. ex Lam.) Urb. | Bombaceae | December-Feb | Tree with long, white, cup-shaped flowers. | Frankie et al. 1974, Janzen and Liesner 1980, Lobo et al. 2008 |

| **Plant Species** | **Family** | **Phenology** | **Brief Description** | **Sources** |
| --- | --- | --- | --- | --- |
| *Palicourea* sp.1 | Rubiaceae | Late April-July | Small tree with long tubular white-pale purple flowers. | Croat 1978 |
| *Palicourea* sp.2 | Rubiaceae | Late April-July | Small tree or shrub with long, tubular, yellow flowers arranged in a panicle. | Croat 1978 |
| *Pentas sp.* | Rubiaceae | Unknown | Shrub with red, star-shaped flowers. |  |
| *Senna alata* (L.) Roxb. | Fabaceae | Dry season, mainly October to March | Shrub with puffy, yellow flowers clustered in racemes. | Frankie et al. 2013 |
| *Stachytarpheta frantzii* Pol. | Verbenaceae | Continuous | Shrub with long tails of purple, small, bell-shaped flowers. | Janzen and Liesner 1980, Croat 1978 |
| *Turnera subulata* | Passifloraceae | Continuous, flowers close every afternoon and open come morning | Subshrub with large white funnel-shaped flowers, and a dark purple center. |  |
| *Zinnia peruviana* (L.) L. | Asteraceae | April-October | Herbaceous plant; with red or yellow composite flowers and with linear or spatulate petals. | Torres 1963 |
| Unknown 1 | Unknown | Unknown | Dark colored vine, with small pink flowers |  |
| Unknown 2 | Unknown | Unknown | Herbaceous with a yellow, composite flowers |  |

**Literature Cited**

Croat, T.B. 1978. Flora of Barro Colorado Island. Standford University Press. Florenwerke, Panama.

Enquist, B. and J. J. Sullivan. 2001. Vegetative key and descriptions of tree species of the tropical dry forests of upland Sector Santa Rosa, Area de Conservación Guanacaste, Costa Rica. 68pp.

Frankie, G. W., H. G. Baker, and P. A. Opler. 1974. Comparative Phenological Studies of Trees in Tropical Wet and Dry Forests in the Lowlands of Costa Rica **62**:881-919.

Frankie, G. W., S. B. Vinson, M. A. Rizzardi, T. L. Griswold, R. E. Coville, M. H. Grayum, L. E. S. Martinez, J. Foltz-Sweat, and J. C. Pawelek. 2013. Relationships of Bees to Host Ornamental and Weedy Flowers in Urban Northwest Guanacaste Province, Costa Rica. Journal of the Kansas Entomological Society **86**: 325-351.

Janzen, D., and R. Liesner. 1980. Annotated check-list of plants of lowland Guanacaste Province, Costa Rica, exclusive of grasses and non-vascular cryptogam. Brenesia **18**: 15-90.

Kriebel, R. 2016. A Monograph of Conostegia (Melastomataceae, Miconieae). Phytokeys **67**:1-326

Lobo, J., R. Aguilar, E. Chacon, and E. Fuchs. 2008. Phenology of tree species of the Osa Peninsula and Golfo Dulce region, Costa Rica.

Singh, D. R., R. P. Medhi, S. Manju, and P. K. Sikdar. 2006. Floral characters of Morinda citrifolia in Bay Islands. International Journal of Noni Research **1**:1-3.

Thomas, R., and A. Rajkumar. 2013. Flowering and pollination biology in coconut. Journal of Plantation Crops **41**:109-111.

Torres, A. M. 1963. Taxonomy of Zinnia. Brittonia **15**: 1-25.

**Table S2.** A list of all invertebrate pollinators collected throughout our study, the total number of visits and which site and year they were collected in.

| **Family** | **Species** | **Number of visits** | **Cañaza** | **Finca Kobo/Palo Seco** | **Mata Palo** | **Osa Conservation** | **Playa Blanca** | **Playa Sandalo** | **Puerto Jimenez** | **Rincon** | **2017** | **2018** | **2019** |
| --- | --- | --- | --- | --- | --- | --- | --- | --- | --- | --- | --- | --- | --- |
| Apidae | *Apis mellifera* | 545 | X | X |  | X | X |  | X | X | X | X | X |
|  | *Bombus pullatus* | 40 |  | X | X | X |  |  | X |  | X | X | X |
|  | *Centris aethiocesta* | 2 |  |  |  |  |  |  | X |  |  | X |  |
|  | *Centris analis* | 17 |  |  |  |  |  |  | X |  |  | X | X |
|  | *Centris dichrootricha* | 2 |  |  |  |  |  |  | X |  |  | X | X |
|  | *Centris nitida* | 2 |  |  |  |  |  |  | X |  |  | X |  |
|  | *Centris obscurior* | 3 |  |  |  |  |  |  | X |  |  | X |  |
|  | *Centris terminata* | 3 |  |  |  |  |  |  | X |  |  |  | X |
|  | *Centris varia* | 4 |  |  |  |  |  |  | X |  |  | X | X |
|  | *Cephalotrigona zexmeniae* | 4 |  |  |  | X |  |  |  | X |  | X | X |
|  | *Ceratina buscki* | 1 |  |  |  |  |  |  | X |  |  | X |  |
|  | *Ceratina chloris* | 281 | X | X | X | X | X | X | X |  | X | X | X |
|  | *Ceratina eximia* | 1 |  |  | X |  |  |  |  |  |  | X |  |
|  | *Ceratina trimaculata* | 1 |  | X |  |  |  |  |  |  |  |  | X |
|  | *Dolichotrigona schulthessi* | 2 |  |  | X |  |  |  |  |  |  | X |  |
|  | *Epicharis albofasciata* | 7 |  |  |  |  |  |  | X |  |  | X |  |
|  | *Euglossa allostica* | 1 |  |  |  | X |  |  |  |  |  | X |  |
|  | *Euglossa atroventa* | 1 |  |  |  |  |  |  | X |  | X |  |  |
|  | *Euglossa azureoviridis* | 9 |  | X |  | X |  |  |  |  | X | X |  |
|  | *Euglossa cyanura* | 4 |  |  |  |  |  |  | X |  |  | X | X |
|  | *Euglossa despecta* | 5 |  |  |  | X |  |  |  |  |  | X |  |
|  | *Euglossa dodsoni* | 4 |  | X |  | X |  |  |  |  |  | X |  |
|  | *Euglossa erythrochlora* | 11 |  |  | X | X |  |  |  |  |  | X |  |
|  | *Euglossa hansoni* | 4 |  |  |  | X |  |  |  |  | X |  |  |
|  | *Euglossa heterosticta* | 8 |  |  |  | X |  |  | X |  | X |  | X |
|  | *Euglossa imperialis* | 4 |  |  |  | X |  |  |  |  | X |  |  |
|  | *Euglossa mixta* | 1 |  |  |  | X |  |  |  |  | X |  |  |
|  | *Euglossa townsendi* | 83 |  | X |  | X |  |  | X |  | X | X | X |
|  | *Euglossa tridentata* | 2 |  |  |  | X |  |  |  |  | X | X |  |
|  | *Euglossa variabilis* | 4 |  | X |  |  |  |  |  |  |  | X | X |
|  | *Euglossa viridissima* | 3 |  |  |  | X |  |  |  |  |  | X |  |
|  | *Eulema cingulata* | 1 |  |  |  | X |  |  |  |  | X |  |  |
|  | *Exaerete dentata* | 1 |  |  |  | X |  |  |  |  | X |  |  |
|  | *Exomalopsis sp. 2* | 2 |  | X |  |  |  |  |  |  |  | X |  |
| **Family** | **Species** | **Number of visits** | **Cañaza** | **Finca Kobo/Palo Seco** | **Mata Palo** | **Osa Conservation** | **Playa Blanca** | **Playa Sandalo** | **Puerto Jimenez** | **Rincon** | **2017** | **2018** | **2019** |
|  | *Exomalopsis sp. 3* | 2 |  | X |  |  |  |  |  |  |  |  | X |
|  | *Exomalopsis sp. 4* | 1 |  |  |  |  |  | X |  |  |  | X |  |
|  | *Exomalopsis sp. 5* | 1 | X |  |  |  |  |  |  |  |  | X |  |
|  | *Melipona fasciata* | 163 | X | X | X | X |  |  |  | X | X | X | X |
|  | *Nannotrigona mellaria* | 107 | X | X |  |  |  | X | X | X | X | X | X |
|  | *Nogueropsis mirandula* | 3 |  |  | X | X |  |  |  |  | X | X |  |
|  | *Oxytrigona mellicolor* | 59 |  | X |  |  |  |  | X |  |  | X | X |
|  | *Paratetrapedia calcarata* | 65 |  | X | X | X | X | X | X | X | X | X | X |
|  | *Paratetrapedia chocoensis* | 14 |  | X | X | X |  |  |  |  | X | X |  |
|  | *Paratetrapedia leucostoma* | 5 |  |  |  | X |  |  | X |  | X |  | X |
|  | *Paratetrapedia moesta* | 3 | X |  |  |  |  |  | X |  |  | X | X |
|  | *Paratetrapedia sp. 2* | 4 |  | X |  | X |  |  | X | X |  | X | X |
|  | *Paratetrapedia volatilis* | 6 |  |  | X | X |  |  |  |  | X | X |  |
|  | *Paratrigona opaca* | 392 | X | X |  | X |  | X | X | X | X | X | X |
|  | *Partamona orizabaensis* | 198 | X | X |  | X |  | X | X | X | X | X | X |
|  | *Plebeia frontalis* | 5 |  |  | X | X |  |  |  |  | X | X |  |
|  | *Plebeia tica* | 7 |  |  | X | X |  |  |  |  | X | X |  |
|  | *Ptilotrigona occidentalis* | 13 |  |  | X | X |  |  |  | X | X | X | X |
|  | *Scaptotrigona pectoralis* | 7 |  |  |  |  |  |  | X |  |  |  | X |
|  | *Scaura latitarsis* | 38 | X | X |  | X |  |  | X | X | X | X | X |
|  | *Tetragona dorsalis* | 72 | X | X | X | X |  | X | X | X | X | X | X |
|  | *Tetragona perangulata* | 53 |  | X |  | X |  |  | X |  | X | X | X |
|  | *Thygater sp. 1* | 4 |  | X |  |  |  |  | X |  |  | X | X |
|  | *Trigona corvina* | 423 | X | X | X | X | X | X | X | X | X | X | X |
|  | *Trigona fulviventris* | 1298 | X | X | X | X | X | X | X | X | X | X | X |
|  | *Trigona fuscipennis* | 271 | X | X |  | X |  |  | X | X | X | X | X |
|  | *Trigona nigerrima* | 30 |  | X |  |  |  |  | X | X | X |  | X |
|  | *Trigona silvestriana* | 27 |  | X |  | X |  |  |  |  | X | X |  |
|  | *Trigonisca atomaria* | 2 |  |  | X | X |  |  |  |  | X | X |  |
|  | *Trigonisca buyssoni* | 3 |  |  | X | X |  |  |  |  | X | X |  |
|  | *Trigonisca discolor* | 1 |  |  |  | X |  |  |  |  |  | X |  |
|  | *Xylocopa ocellaris* | 3 |  |  |  | X |  |  |  |  | X |  |  |
| Halictidae | *Agapostemon sp. 1* | 2 |  |  |  |  |  |  | X |  |  |  | X |
|  | *Augochlora sp. 1* | 13 | X | X |  | X |  |  | X |  | X | X |  |
|  | *Augochlora sp. 12* | 1 |  |  |  |  |  |  |  | X |  |  | X |
|  | *Augochlora sp. 13* | 1 |  |  |  | X |  |  |  |  | X |  |  |
| **Family** | **Species** | **Number of visits** | **Cañaza** | **Finca Kobo/Palo Seco** | **Mata Palo** | **Osa Conservation** | **Playa Blanca** | **Playa Sandalo** | **Puerto Jimenez** | **Rincon** | **2017** | **2018** | **2019** |
|  | *Augochlora sp. 14* | 1 |  |  |  | X |  |  |  |  | X |  |  |
|  | *Augochlora sp. 15* | 17 |  | X |  | X |  |  |  | X |  | X | X |
|  | *Augochlora sp. 18* | 54 | X | X |  | X |  |  | X | X | X | X | X |
|  | *Augochlora sp. 19* | 103 | X | X |  | X |  |  | X |  | X | X | X |
|  | *Augochlora sp. 20* | 6 | X | X |  |  |  |  | X |  | X | X |  |
|  | *Augochlorini sp. 2* | 1 |  |  |  | X |  |  |  |  | X |  |  |
|  | *Augochlorini sp. 3* | 9 | X | X |  | X |  |  |  | X | X | X | X |
|  | *Augochloropsis sp. 2* | 20 | X | X |  | X |  |  |  |  | X | X | X |
|  | *Augochloropsis sp. 3* | 21 | X | X |  | X |  |  |  | X |  | X | X |
|  | *Augochloropsis ignita* | 29 | X | X |  | X |  |  |  | X | X | X | X |
|  | *Augochloropsis sp. 5* | 29 | X | X |  |  |  | X |  | X |  | X | X |
|  | *Halictini sp. 5* | 2 | X | X |  |  |  |  |  |  |  | X |  |
|  | *Lasioglossum (dialictus) sp. 1* | 39 | X | X | X | X |  |  | X | X | X | X | X |
|  | *Lasioglossum (dialictus) sp. 5* | 1 |  | X |  |  |  |  |  |  | X |  |  |
|  | *Psuedoaugochlora graminea* | 199 | X | X | X | X |  | X | X |  | X | X | X |
| Megachilidae | *Megachile sp. 1* | 1 |  |  |  | X |  |  |  |  |  | X |  |
|  | *Megachile sp. 5* | 2 |  |  |  | X |  |  |  |  |  | X |  |
| Hesperiidae | *Autochton bipunctatus* | 1 |  | X |  |  |  |  |  |  |  |  | X |
|  | *Autochton sp. 1* | 4 |  | X |  |  | X |  |  |  |  |  | X |
|  | *Bolla sp.* | 1 |  |  |  |  |  |  |  | X |  |  | X |
|  | *Chioides catillus* | 55 | X | X |  | X | X |  | X | X | X | X | X |
|  | *Hesperiidae sp. 4* | 1 |  |  |  |  |  |  | X |  |  |  | X |
|  | *Hesperiidae sp. 1* | 5 |  | X |  | X |  |  |  |  | X | X | X |
|  | *Hesperiidae sp. 10* | 6 |  |  |  | X |  |  | X |  | X | X | X |
|  | *Hesperiidae sp. 11* | 3 | X |  |  | X |  |  |  |  | X | X |  |
|  | *Hesperiidae sp. 12* | 11 |  |  | X | X |  | X | X |  | X | X |  |
|  | *Hesperiidae sp. 13* | 2 |  |  |  | X |  |  |  |  |  | X |  |
|  | *Hesperiidae sp. 14* | 46 | X | X | X |  | X |  | X | X |  | X | X |
|  | *Hesperiidae sp. 16* | 2 | X |  | X |  |  |  |  |  |  | X |  |
|  | *Hesperiidae sp. 2* | 10 |  |  |  | X | X |  |  |  | X | X | X |
|  | *Hesperiidae sp. 3* | 3 |  |  |  | X |  |  |  |  | X | X |  |
|  | *Hesperiidae sp. 4* | 10 |  |  |  | X |  |  |  |  | X |  |  |
|  | *Hesperiidae sp. 6* | 1 |  |  |  | X |  |  |  |  | X |  |  |
| **Family** | **Species** | **Number of visits** | **Cañaza** | **Finca Kobo/Palo Seco** | **Mata Palo** | **Osa Conservation** | **Playa Blanca** | **Playa Sandalo** | **Puerto Jimenez** | **Rincon** | **2017** | **2018** | **2019** |
|  | *Hesperiidae sp. 7* | 8 |  |  |  | X |  |  | X |  | X | X | X |
|  | *Hesperiidae sp. 8* | 8 |  | X |  | X | X |  |  | X | X |  | X |
|  | *Hesperiidae sp. 9* | 1 |  |  |  | X |  |  |  |  | X |  |  |
|  | *Hylephila phyleus* | 1 |  |  |  |  |  |  | X |  |  |  | X |
|  | *Morys micythus* | 1 |  |  |  |  |  |  | X |  |  |  | X |
|  | *Niconiades nikko* | 1 |  |  |  |  |  |  | X |  |  |  | X |
|  | *Polythrix caunus* | 1 |  | X |  |  |  |  |  |  |  |  | X |
|  | *Polythrix sp. 2* | 1 |  | X |  |  |  |  |  |  |  |  | X |
|  | *Proteides sp. 1* | 1 |  |  |  |  |  |  | X |  |  |  | X |
|  | *Pyrgus sp. 1* | 6 | X |  |  | X |  | X | X | X | X | X | X |
|  | *Thorybes drusius* | 1 |  |  |  |  | X |  |  |  |  |  | X |
|  | *Urbanus albimargo* | 2 |  | X |  |  |  |  | X |  |  |  | X |
|  | *Urbanus dorantes* | 22 | X |  |  |  | X |  | X | X |  | X | X |
|  | *Urbanus doryssus* | 1 |  |  |  |  |  |  | X |  |  |  | X |
|  | *Urbanus esmeraldus* | 1 |  | X |  |  |  |  |  |  |  |  | X |
|  | *Urbanus procne* | 2 |  |  |  |  |  |  | X |  |  |  | X |
|  | *Urbanus proteus* | 11 |  | X |  |  | X |  | X |  |  |  | X |
|  | *Urbanus simplicius* | 49 |  | X |  |  | X |  | X | X |  |  | X |
|  | *Urbanus tanna* | 5 |  | X |  |  |  |  | X |  |  |  | X |
|  | *Urbanus teleus* | 25 |  |  |  | X | X |  | X |  | X | X | X |
|  | *Urbanus viterboana* | 10 |  |  |  | X |  |  |  |  | X | X |  |
| Lycaenidae | *Cyanophrys fusius* | 1 |  |  |  | X |  |  |  |  | X |  |  |
|  | *Declinea percosius* | 1 |  | X |  |  |  |  |  |  |  |  | X |
|  | *Leptotes cassius* | 2 |  |  |  |  |  | X |  |  |  | X |  |
|  | *Lycaenidae sp. 1* | 1 |  |  |  | X |  |  |  |  |  | X |  |
|  | *Pseudolycaena sp.* | 1 |  |  |  |  |  |  | X |  |  |  | X |
|  | *Strymon ziba* | 1 |  |  |  |  |  |  | X |  |  |  | X |
| Nymphalidae | *Adelpha cytherea* | 1 |  |  |  |  |  | X |  |  |  | X |  |
|  | *Agraulis vanillae* | 11 |  |  |  |  | X |  |  |  |  |  | X |
|  | *Anartia fatima* | 170 | X | X | X | X | X |  | X | X | X | X | X |
|  | *Anartia jatrophae* | 42 | X | X |  | X | X | X | X |  | X | X | X |
|  | *Chlosyne ezra* | 4 |  |  |  |  |  |  | X |  |  |  | X |
|  | *Cissia hermes* | 8 |  | X | X | X |  |  | X |  | X | X | X |
|  | *Dione juno* | 1 |  |  |  | X |  |  |  |  |  | X |  |
|  | *Dryadula phatusa* | 1 |  |  |  |  |  |  | X |  |  |  | X |
|  | *Dryas iulia* | 8 | X |  |  |  |  |  | X |  |  | X | X |
|  | *Euptoieta claudia* | 4 |  |  |  |  | X |  | X |  |  |  | X |
| **Family** | **Species** | **Number of visits** | **Cañaza** | **Finca Kobo/Palo Seco** | **Mata Palo** | **Osa Conservation** | **Playa Blanca** | **Playa Sandalo** | **Puerto Jimenez** | **Rincon** | **2017** | **2018** | **2019** |
|  | *Euptoieta hegesia* | 33 | X | X |  | X |  |  | X |  |  | X | X |
|  | *Heliconius cydno* | 2 |  |  |  | X |  |  |  |  | X |  |  |
|  | *Heliconius erato* | 22 |  |  | X | X |  |  | X |  | X | X | X |
|  | *Heliconius hecale* | 10 |  |  |  | X |  |  |  |  | X |  |  |
|  | *Heliconius hecalesia* | 1 |  |  |  | X |  |  |  |  | X |  |  |
|  | *Heliconius hewitsoni* | 14 |  |  | X | X |  |  |  |  | X | X |  |
|  | *Heliconius ismenius* | 30 | X | X |  | X | X |  | X |  | X | X | X |
|  | *Heliconius melpomene* | 6 | X |  |  | X |  |  |  |  | X | X |  |
|  | *Heliconius pachinus* | 12 |  |  | X | X |  |  |  |  |  | X |  |
|  | *Heliconius theudela* | 1 |  |  |  |  |  |  |  | X |  |  | X |
|  | *Hermeuptychia sosybius* | 1 |  | X |  |  |  |  |  |  |  |  | X |
|  | *Junonia evarete* | 4 |  | X |  | X |  |  |  |  | X |  | X |
|  | *Junonia genoveva* | 3 |  | X |  |  |  |  | X |  |  |  | X |
|  | *Thessalia ezra* | 1 |  |  |  |  |  |  | X |  |  |  | X |
| Papilionidae | *Battus polydamos* | 1 |  |  |  |  |  |  | X |  |  |  | X |
|  | *Heraclides thoas* | 2 |  |  |  | X |  |  |  |  | X |  |  |
| Pieridae | *Aphrissa boisduvalii* | 5 |  |  |  |  |  |  | X | X |  |  | X |
|  | *Aphrissa statira* | 3 |  | X |  |  |  |  | X |  |  |  | X |
|  | *Appias drusilla* | 2 |  |  |  |  |  |  | X |  |  |  | X |
|  | *Ascia monuste* | 26 | X | X |  | X |  |  | X | X | X | X | X |
|  | *Eurema albula* | 1 |  | X |  |  |  |  |  |  |  |  | X |
|  | *Eurema daira* | 3 |  | X |  |  |  | X |  |  |  | X | X |
|  | *Eurema nise* | 6 | X |  |  | X |  |  | X |  | X | X | X |
|  | *Eurema proterpia* | 2 |  |  |  |  | X |  | X |  |  |  | X |
|  | *Phoebis agarithe* | 1 |  |  |  |  |  |  | X |  |  |  | X |
|  | *Phoebis argante* | 2 |  | X |  |  |  |  | X |  |  |  | X |
|  | *Phoebis philea* | 1 |  |  |  |  |  |  | X |  |  |  | X |
|  | *Phoebis sennae* | 35 |  | X | X | X | X |  | X | X | X | X | X |
| Riodinidae | *Nymphidium ascolia* | 1 |  |  |  | X |  |  |  |  | X |  |  |
|  | *Rhetus arcius* | 1 |  |  |  | X |  |  |  |  |  | X |  |
|  | *Theope publius* | 1 |  |  |  | X |  |  |  |  | X |  |  |

**Table S3.** Observed species richness and estimated asymptotic richness of the visiting insect pollinator assemblage for each plant species with ten or more samples, as well as the calculated percentage of maximum estimated species detected within our study for each plant species (%S_O_)(see Chacoff et al. 2012).

| **Plant Species** | **Number of Samples** | **Observed Species Richness (S_O_)** | **Asymptotic Richness (S_E_±SE)** | **Percent Detected**  **(%S_O_ = 100**$\frac{\mathbf{S}_{\mathbf{O}}}{\mathbf{S}_{\mathbf{E}}}$ **)** |
| --- | --- | --- | --- | --- |
| *Caesalpinia pulcherrima* | 78 | 43 | 116 ± 53.2 | 37.1 |
| *Celosia argentea* | 13 | 32 | 39 ± 5.1 | 82.1 |
| *Conostegia subcrustulata* | 77 | 41 | 325 ± 307.5 | 12.6 |
| *Cornutia grandifolia* | 31 | 29 | 34 ± 4.5 | 85.3 |
| *Hamelia patens* | 96 | 36 | 55 ± 13.0 | 65.5 |
| *Isertia haenkeana* | 19 | 13 | 25 ± 12.5 | 52.0 |
| *Lantana camara* | 33 | 41 | 88 ± 29.8 | 46.6 |
| *Musa sp.* | 25 | 12 | 36 ± 29.9 | 33.3 |
| *Palicourea sp. 2* | 17 | 22 | 39 ± 13.6 | 56.4 |
| *Stachytarpheta frantzii* | 103 | 90 | 128 ± 18.0 | 69.5 |
| *Turnera subulate* | 47 | 44 | 72 ± 18.9 | 59.7 |
| *Zinnia peruviana* | 18 | 36 | 74 ± 24.7 | 48.7 |

**Table S4.** Species level attributes for all plants within the full network

| **Plant Species** | **Degree** | **Normalized Degree** | **Species Strength** | **Weighted Betweenness** | **Closeness Centrality** |
| --- | --- | --- | --- | --- | --- |
| *Arachis pintoi* | 9 | 0.05 | 3.30 | 0 | 0.01 |
| *Caesalpinia pulcherrima* | 43 | 0.25 | 13.27 | 0.19 | 0.11 |
| *Celosia argentea* | 32 | 0.19 | 6.56 | 0.0 | 0.04 |
| *Cocos nucifera* | 9 | 0.05 | 3.03 | 0.0 | 0.04 |
| *Conostegia subcrustulata* | 41 | 0.24 | 14.25 | 0.24 | 0.06 |
| *Cornutia grandifolia* | 29 | 0.17 | 11.62 | 0 | 0.03 |
| *Cosmos sulphureus* | 8 | 0.05 | 1.46 | 0 | 0.02 |
| *Hamelia patens* | 36 | 0.21 | 8.0 | 0.27 | 0.12 |
| *Isertia haenkeana* | 13 | 0.08 | 2.13 | 0 | 0.03 |
| *Lantana camara* | 41 | 0.24 | 10.38 | 0 | 0.05 |
| *Miconia lacera* | 7 | 0.04 | 1.47 | 0 | <0.01 |
| *Morinda citrifolia* | 1 | <0.01 | 0.08 | 0 | <0.01 |
| *Musa sp.* | 12 | 0.07 | 1.37 | 0.05 | 0.05 |
| *Ochroma pyramidale* | 8 | 0.05 | 1.85 | 0 | <0.01 |
| *Palicourea sp.* | 3 | 0.02 | 0.16 | 0 | <0.01 |
| *Palicourea sp. 2* | 22 | 0.13 | 2.78 | 0 | 0.02 |
| *Pentas sp.* | 5 | 0.03 | 0.78 | 0 | 0.01 |
| *Senna alata* | 5 | 0.03 | 0.12 | 0 | <0.01 |
| *Stachytarpheta frantzii* | 89 | 0.52 | 54.19 | 0 | 0.07 |
| *Unknown 1* | 2 | 0.01 | 0.01 | 0 | <0.01 |
| *Unknown 2* | 10 | 0.06 | 3.16 | 0 | <0.01 |
| *Allamanda cathartica* | 1 | <0.01 | <0.01 | 0 | <0.01 |
| *Bauhinia monandra* | 1 | <0.01 | <0.01 | 0 | <0.01 |
| *Turnera subulata* | 43 | 0.25 | 18.62 | 0.26 | 0.12 |
| *Zinnia peruviana* | 36 | 0.21 | 12.41 | 0 | 0.04 |

**Figures**

**Figure S1.** Map of the study site: Osa Peninsula, Costa Rica. Yellow blocks indicate the eight sampling locations.

**Figure S2.** Accumulation curves of a) species and b) interactions as sampling effort increases with all plant species pooled. Horizontal lines represent asymptotic richness (solid line) and standard error (dashed lines).

**Figure S3.** Abbreviated modularity plot illustrating the partitioning of our full network into modules when optimum modularity was reached. All interactions were plotted within this matrix but condensed into a proportion of plant-pollinator interactions for figure clarity. Interactions are represented by the shaded blue squares; darker shadings indicate stronger interactions. Red boxes depict what plants and pollinators belong to the same module.
